# Influence of Noise-Limited Censored Path Loss on Model Fitting and Path Loss-Based Positioning †

**DOI:** 10.3390/s21030987

**Published:** 2021-02-02

**Authors:** Aki Karttunen, Mikko Valkama, Jukka Talvitie

**Affiliations:** Faculty of Information Technology and Communication Sciences, Tampere University, 33014 Tampere, Finland; mikko.valkama@tuni.fi (M.V.); jukka.talvitie@tuni.fi (J.T.)

**Keywords:** positioning, path loss, path loss model, maximum-likelihood estimation, censored data, localization, shadow fading, wireless networks, probabilistic modeling

## Abstract

Positioning is considered one of the key features in various novel industry verticals in future radio systems. Since path loss (PL) or received signal strength-based measurements are widely available in the majority of wireless standards, PL-based positioning has an important role among positioning technologies. Conventionally, PL-based positioning has two phases—fitting a PL model to training data and positioning based on the link distance estimates. However, in both phases, the maximum measurable PL is limited by measurement noise. Such immeasurable samples are called censored PL data and such noisy data are commonly neglected in both the model fitting and in the positioning phase. In the case of censored PL, the loss is known to be above a known threshold level and that information can be used in model fitting and in the positioning phase. In this paper, we examine and propose how to use censored PL data in PL model-based positioning. Additionally, we demonstrate with several simulations the potential of the proposed approach for considerable improvements in positioning accuracy (23–57%) and improved robustness against PL model fitting errors.

## 1. Introduction

### 1.1. Motivation

Radio-based positioning has rapidly grown into one of the most significant features in future wireless networks. As stated in the specifications of the upcoming 5th generation of new radio networks in [1], positioning is considered part of basic network capability, and it offers a wide variety of performance requirements tailored to specific needs of numerous use cases and industry verticals. Path loss (PL) or received signal strength (RSS)-based positioning, studied earlier, for example, in [2,3,4,5,6,7,8], enables low-cost positioning capability. PL-based positioning is especially useful for use cases with limited power and computational resources. In addition, PL-based positioning can introduce additional support to various other high-precision positioning and tracking solutions. As shown, for example, in [8,9] PL or RSS positioning can provide increased positioning accuracy, availability, stability or reliability when combined with other methods. Since PL and RSS are power-related measurements, they are typically continually measured and monitored in mobile networks over multiple base stations (BS) to support mobility management and other radio resource management functionalities. Thus, PL-based positioning can rely on regular reference signals of the underlying communications system without introducing any additional training overhead due to positioning capability. However, due to a challenging and highly dynamic propagation environment, PL-based positioning methods are typically limited to positioning accuracy of tens or hundreds of meters in outdoor cellular networks [3,5,6].

**Figure 1:**
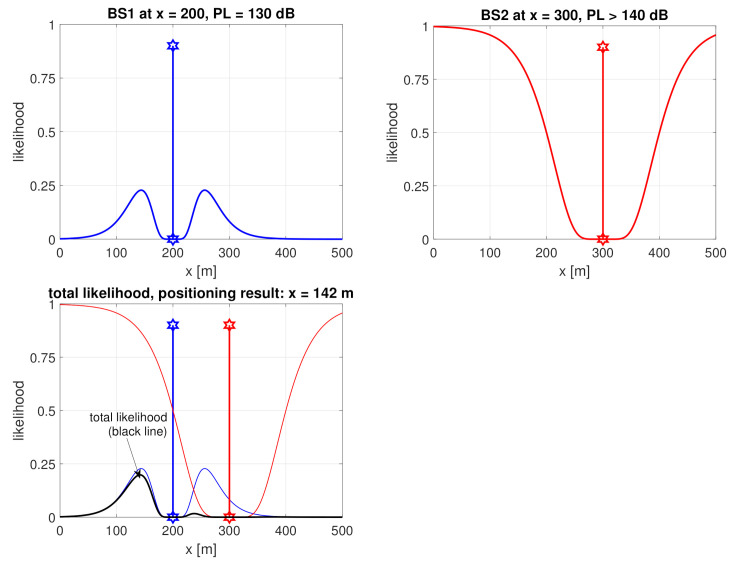
Likelihood function illustration example in 1D: measured path loss (PL) of 130 dB to BS1, censored PL >140 dB to BS2, and the combined likelihood function. Note that one measured and one censored PL are sufficient to get a unique positioning solution and the same is true in 2D and 3D.

Conventionally PL-based positioning has two phases (e.g., [3,4,5]): (i) fitting a PL model to training data, if such training data are available, and (ii) determining link distance estimates based on the PL model and calculating the position estimate. Channel measurements can be used to measure the training data and then a PL model can be fitted to the data [3,10,11,12,13]. The PL model describes the link distance dependency and the variation from the expected value, i.e., shadow fading (SF). Sometimes training data may be unavailable and the PL model can be taken from a standard channel model, e.g., [14]. In practice, the maximum measurable PL is limited by measurement noise. Therefore, in both the training data and in the positioning it may happen that the PL value cannot be determined. In that case, the PL is known to be more than the noise threshold level. When data above or below a certain range are immeasurable, meaning that all data above or below a certain range are counted, but not measured, the term is censored data [10,11,13,15,16,17,18]. The PL model can be fitted to censored data by using Tobit maximum likelihood estimation (MLE) [10,11,13]. Just as in the training phase, the noise threshold limits the maximum PL in the positioning phase. When PL measured from a certain BS is larger than the noise threshold, the true location is likely to be far from the BS, and the true distance is subject to the distance dependence of the PL and the threshold level. Therefore, measuring PL at the positioning phase is censoring data in the same fashion as in the case of the training data. Censored PL can be taken into account in the likelihood function in the positioning phase [3,13,19,20,21,22,23]. Likelihood function examples of a measured and censored PL are presented in Figure 1.

### 1.2. Contributions

The censored PL data (may) exist in both the training phase and the positioning phase. Therefore, in terms of taking or not taking into account the censored samples, there are four options. In [10], fitting without the censored data is called ordinary least squares (OLS) fitting, as opposed to the Tobit MLE. Similarly, for the positioning there is the ordinary positioning, using only the distance estimates from the contacted BSs, which we now call ordinary trilateration positioning (OTP). Then, there is the option to include the censored data with Tobit MLE positioning. The four options are [13]:

- OLS-OPT, with ordinary least squares fitting to the training data and the ordinary trilateration positioning.
- MLE-OPT, with Tobit MLE fitting to the training data and the ordinary trilateration positioning.
- OLS-MLE, with ordinary least squares fitting to the training data and the Tobit MLE positioning.
- MLE-MLE, with Tobit MLE fitting to the training data and the Tobit MLE positioning.

Traditionally, distance estimates between two sources in one dimension (e.g., corridor), three sources in two dimensions (e.g., antennas at about the same height), or four sources in the typical three dimensions, are needed to give a unique position estimate. When the censored PLs are included, this requirement is loosened significantly, as only one contacted and one censored source are needed to give the unique position estimate. The one contacted BS provides one distance estimate (two points in 1D, circle in 2D, or sphere in 3D) and the censored BS selects the furthest point as the unique position estimate. An example in 1D is presented in Figure 1.

To the best of the authors’ knowledge, this is the first time (1) the effect of the noise-limited censored path loss data has been studied in both phases of the PL model-based positioning and both phases are explicitly written as Tobit MLE; (2) a selection criterion accounting for BSs with censored PL in the positioning phase is presented; and (3) the reduced amount of information needed for a unique positioning solution is noted. We examine the influence of including the censored data through simple simulations. The simulations use a typical log-distance PL model and illustrate the potential of the proposed approach for considerable improvements in positioning accuracy. Three different realistic PL distributions are considered, including examples in which line-of-sight (LOS) and non-line-of-sight (NLOS) follow different distributions according to [14]. Additionally, the dependence on the noise threshold level and LOS detection probability is examined. Usually, in positioning the antennas are, or are assumed to be, omnidirectional [2,3,4,5,6,7,8,9,19,20,21,22,23]. In this paper, the positioning simulations compare omnidirectional and directional BS antennas assuming a simple antenna model.

### 1.3. Extension

This paper is an extended version of the authors’ conference paper [13]. In this extended paper, we present more examples and a more extensive analysis demonstrating the influence of the censored PL on positioning in a wide range of radio channel conditions. The conference paper is extended as follows: (1) more examples with realistic PL distributions, one with the model parameters taken from a measurement campaign and one using the 3GPP path loss model for urban microcellular scenarios at 2 and 28 GHz; (2) a selection criterion for the BSs with censored PL; (3) the reduced amount of information needed for a unique positioning solution is noted; (4) examples with different LOS and NLOS PL distributions; (5) examination of the influences of the noise threshold value and LOS detection probability.

### 1.4. Organization of This Paper

The remainder of the paper is organized as follows: The noise-limited PL is explained in more detail in Section 2. Section 3 lists the path loss, antenna models, and three examples of PL distributions used in this study. In Section 4, the PL model is fitted to simulated training data. The PL model fitting results are in Section 5. Positioning with or without the censored PL is examined in Section 6 and the positioning simulation results are presented in Section 7. Finally, conclusions are given in Section 8.

## 2. Noise-Limited Path Loss and Positioning

In this section, we present some definitions and prior works related to PL-based positioning. These include the definitions of RSS and PL, the determination of a PL value, and a review of prior works on the two phases of PL-based positioning with noise-limited PL (or RSS). The PL and antenna models used in this study are then presented in Section 3.

Path loss is the inverse of small-scale-averaged path gain between the base station (BS) and the mobile station (MS) calculated as the instantaneous local channel gain averaged over the small-scale fading. The positioning can be done based on RSS or PL. Provided that the RSS is small-scale averaged, the only relevant difference is that for PL the transmit power needs to be known. In both cases, the measured RSS or PL is noise limited, and it is therefore censored data. Noise-limited RSS is under the receiver noise threshold level and noise-limited PL is over the path loss noise threshold. In this paper, the notation is given for PL, but the methods and equations apply also for RSS positioning with minor changes.

The instantaneous local channel gain is the difference between transmitted and received power, and importantly, it includes the small-scale fading, i.e., multipath fading. For PL, the small-scale-averaging can be performed by averaging over traveled distance, time, antenna elements, or frequency. If the multipath powers can be resolved, the PL can be calculated as the inverse of sum of the multipath powers. Measurement of RSS or the multipath powers is limited by noise which affects the PL values in case of poor or limited signal to noise ratio (SNR). Therefore, even when calculating a single PL value the data are truncated [24] or censored [25]. Reference [24] examines how to calculate PL from instantaneous RSS that is truncated by noise. In [25], the detectable multipaths are limited by the measurement noise, and therefore, the sum of the multipath powers is censored data. Naturally, a PL measurement can also be unaffected by noise when the SNR is good or simply censored by the noise power when the measurement is practically only noise. For simplicity, in this paper, we assume that the PL values are either available or censored by noise.

Censored PL samples are typically ignored as outages and are not taken into account, which can lead to significant error in the PL model distribution. The censored PL samples can be taken into account when fitting the PL model to the training data using Tobit MLE [10,11,13]. For example, in [10] it is shown with measured path loss data that by ignoring the censored samples the slope of the PL model, i.e., path loss exponent, is drastically underestimated at 1.3 instead of 2.2. Thus, it is important to use the Tobit MLE when fitting the model to the training data. Note that ignoring the censored PL data in the training phase may lead to a similarly erroneous PL model as when getting the model from the literature without conducting the laborious training measurements.

In the training data, there are two types of data samples—ones with measured PLs and those with PLs larger than the threshold. In the positioning phase, there are two types of data samples—ones with distance estimates and ones with distance estimates more than a threshold distance. The noise-limited measurements can be taken into account in the positioning phase [3,13,19,20,21,22,23]. In [20,22,23], the likelihood of connecting or not connecting to a BS is taken into account in the case of time-of-arrival (TOA) positioning. In [20], it is noted that the audibility information resolves most of the ambiguity when a unique solution is not available with less than three signals measured in 2D. Reference [19] proposes a likelihood function for failing to detect a device in the case when the received power is below a threshold value. The noise level is taken into account in the likelihood function using proximity and quantized RSS positioning in [21]. Two-phased RSS-based positioning method is presented in [3]. In [3], the data are binned and truncated, i.e., rounded to the closest dBm and only a limited number of strongest signals are available, and the incomplete data are taken into account in both phases. In this paper, unlike in [3,19,20,21,22,23], we frame both the training and the positioning phase as Tobit MLE, and we examine the influence of including or not including the censored PL in both phases.

It should be noted that (nearly) all positioning methods have the potential to include the noise-limited censored data. In this work, we focus on the probabilistic PL positioning since censored PL has a simple interpretation as the probability of measuring only noise. Any measurement, e.g., Doppler and angle-of-arrival (AoA) [26], not just PL or RSS, is potentially unavailable due to noise and the likelihood that a censored measurement could in principle be included. Notably, at a relatively short distance, e.g., in case of neighbor discovery [27], censoring does not happen. Additionally, e.g., in wireless sensor networks (WNSs) [28,29] positioning is based on measuring link distance estimates with RSS and/or TOA. As such measurements are limited by noise, the availability or unavailability has a similar link distance-dependent probability as does censored PL.

## 3. Path Loss and Antenna Model

A PL model describes the probability distribution as a function of the link distance, and possibly, also as a function of frequency, BS height, etc. The model has two parts—a model for the expected value and a model for SF, i.e., the variation from the expected value. The expected value is typically modeled as a simple linear function of the logarithm of the link distance and the SF is modeled as a zero-mean log-normal distribution. If needed, the SF model may include the auto-correlation function and correlation distance. Usually, the standard deviation of the SF, σ, is assumed to be a constant, e.g., [14], but it can be also a function of the link distance [11]. In channel models, such as [14,30], there are two models, one for the line-of-sight (LOS) scenario and one for the non-line-of-sight (NLOS) scenario, depending on whether the line-of-sight is clear of obstacles. In positioning, typically there is no distinction between LOS and NLOS, and the same model is used for both—e.g., [2,3,4,5,6,7,8,9,13,19,21,22,23].

Path loss-based positioning always has two PL distributions that may be more or less different. These are the true PL distribution and the PL model fitted to training data and used in the positioning to estimate the link distances based on the measured PL to the BSs. The purpose of the training phase is to attain accurate information on the PL distribution. In practice, there are several possible reasons why the model used in the positioning phase may either underestimate or overestimate the link distances as compared to the true PL distribution. In this paper, we examine one such reason, namely, the effect of ignoring the noise-limited censored PL data.

A simple log-distance PL model is assumed as follows:

(1) $$PL\left(d\right)=\bar{PL}\left(d\right)+S,$$

(2) $$\bar{PL}\left(d\right)=10\cdot \alpha \cdot \log_{10}\left(d/d_{0}\right)+\beta ,$$

where *S* is the zero-mean shadow fading with variance $\sigma^{2}$, $\bar{PL}\left(d\right)$ is the link distance-dependent expected path loss, and $d_{0}=1$ m is a reference distance. Equation (2) has two free parameters: path loss exponent α and floating intercept point β, which can be interpreted as the mean PL at $d_{0}$. Parameters α, β, and σ can be attained by fitting the model to measurement data or, e.g., taken from a channel model. Additionally, other PL models exist, such as the close-in (CI) reference model, where β is fixed to free-space path loss at the reference distance [31,32], and the dual slope model in which the path loss exponent changes after a break point [11,14]. In this work, we use the single-slope log-distance PL model (1) and (2) when fitting the model to training data and in the positioning phase. It should be noted that the methods presented in this paper are not limited only to the single-slope model.

Models such as [14] also define SF correlation properties. SF inter-site cross-correlation is small for widely spaced sites and large for closely spaced BSs [33,34]. Therefore, we use zero correlation between BS sites and the same SF for different beams of the same BS location. In this work, we do not use any tracking algorithm, and therefore SF auto-correlation function and correlation distance are not defined.

In the case of a directive antenna, we approximate

**Figure 2:**
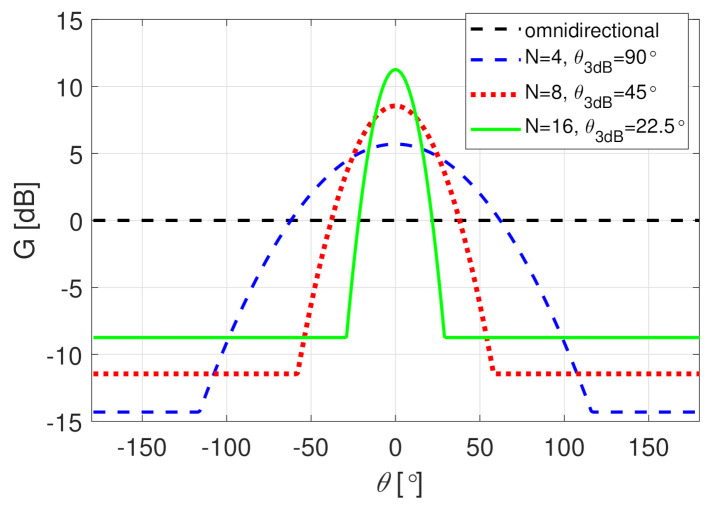
Antenna gain as a function of the offset angle θ. The directive beam patterns have beam-width of $\theta_{3\mathrm{dB}}=360^{\circ }/N$.

(3) PL(d,θ)=PL(d)−G(θ),

(4) $$PL\left(d,\theta \right)=10\cdot \alpha \cdot \log_{10}\left(d/d_{0}\right)+\beta -G\left(\theta \right)+S,$$

where G(θ) is the antenna gain pattern. A simple BS antenna pattern from [14,35] is used:

(5) $$A\left(\theta \right)=-\min(12{\left(\theta /\theta_{3\mathrm{dB}}\right)}^{2},20\;\mathrm{dB}),$$

where min(·) denotes the minimum function, θ is the offset angle from boresight, and $\theta_{3\mathrm{dB}}$ is the antenna half-power beam-width. The relative sidelobe level is fixed at a constant 20 dB below the maximum gain. Antenna gain G(θ) is the A(θ) normalized for unit gain, i.e., for same total radiated power as with an omni-directional antenna with G(θ)=0 dB. The BS is assumed to cover 360${}^{\circ }$ with *N* antenna beams with 3 dB beam overlap, i.e., $\theta_{3\mathrm{dB}}=360^{\circ }/N$. Examples of the omnidirectional and directive antenna patterns are illustrated in Figure 2. Only the omni-directional and 8-sector BS antennas (N=8, $\theta_{3\mathrm{dB}}=45^{\circ }$, max(G)=9 dB) are taken as examples in this paper. The MS has omni-directional antenna. The approximation (3) assumes that (most of) the power is near the direct line between BS and MS. More accurately, the antenna gain is applied to the multipaths that may arrive/depart at any angle [14]. Additionally, the user effect, e.g., [36,37,38], and polarization of antennas and the radio channel, e.g., [18], are ignored in this study. Nevertheless, the simplistic approximation is assumed, as it allows simple simulations with directive antennas to examine the influence of the noise-limited censored PL.

In the simulations conducted in this study, we considered three example PL distributions that were used to create the true PL samples in both training and positioning phases. These examples were:

- Example 1, in which, the parameter values are selected as approximate median values given in 3GPP model [14] for various environments including both LOS and NLOS in both outdoor and indoor environments. For simplicity we do not distinguish between LOS and NLOS, nor do we specify the used radio frequency. The model (1) and (2) is used with parameters α=4, β=60 dB, σ=7 dB, and noise threshold at 140 dB [13].
- Example 2 is similar to Example 1, except that the parameter values were taken from a channel measurement campaign [10]. In [10], the parameters, when the censored PL data points are taken into account, are α=2.2, β=51 dB, σ=7.6 dB, and noise threshold at 95 dB.
- Example 3, the third example is the 3GPP path loss model for urban microcellular scenarios at 2.0 GHz and 28 GHz frequencies [14]. The model is a dual-slope model with different parameters for LOS and NLOS. The LOS-state is defined by a link distance-dependent LOS probability model.

In all of the examples, we assume that the PL statistics are stationary and use the same PL model for all BSs [5,12,30,39]. The BS antenna is either an omni-directional or an eight-sector directive antenna that covers 360${}^{\circ }$ with $\theta_{3\mathrm{dB}}=45^{\circ }$ and max(G)=9 dB. The model-fitting results are presented in Section 5 and the positioning simulations in Section 7.

## 4. Censored Path Loss and Model Fitting

The first phase of the two-phase PL model-based positioning is the measurement of training data and fitting the model to the data. Training data consist of $N_{s}$ samples of PL and the corresponding linked distances. In OLS fitting, only these measurable PL samples are used. In the case of noise-limited censored PL, the link distance is known and PL is more than the threshold limit $PL^{*}$. Using Tobit MLE, the censored PL samples can be taken into account [10,11,13].

The likelihood of measuring PL is [10]

(6) $$l\left(PL\right)=\left(1/\sigma \right)\phi (\left(PL-\bar{PL}\right)/\sigma ),$$

where σ is the standard deviation (std) of the shadow fading, and $\bar{PL}$ is the expected path loss model. Here, ϕ(·) is the standard normal probability density function (PDF). The log-likelihood function for known PL samples at distances $d_{i}$ is

(7) $$LLF=\sum_{i=1}^{N_{s}}\left(-\ln\sigma +\ln\phi \left(\frac{PL\left(d_{i}\right)-\bar{PL}\left(d_{i}\right)}{\sigma }\right)\right),$$

where $N_{s}$ is the number of uncensored data samples. The likelihood of measuring $PL>PL^{*}$ is [10]

(8) $$l\left(PL>PL^{*}\right)=1-\Phi \left(\left(PL^{*}-\bar{PL}\right)/\sigma \right),$$

where Φ(·) is the cumulative distribution function (CDF) of the standard normal distribution. The log-likelihood function for censored samples at distances $d_{i}$ is

(9) $$LLF^{*}=\sum_{i=1}^{N_{s}^{*}}\ln\left(1-\Phi \left(\frac{PL^{*}-\bar{PL}\left(d_{i}\right)}{\sigma }\right)\right),$$

where ${}^{*}$ refers to censored data; i.e., $N_{s}^{*}$ is the number of censored data points.

The path loss parameters are then estimated as the minimum of the negative of the log-likelihood function. For the OLS fitting, the censored samples are not used; i.e.,

(10) $$\left[\hat{\alpha },\hat{\beta },\hat{\sigma }\right]=\underset{}{\arg}\min_{\alpha ,\beta ,\sigma }\left\{-LLF\right)\},$$

where parameter estimates are marked with $\hat{\cdot }$; i.e., $\hat{\alpha }$, $\hat{\beta }$, and $\hat{\sigma }$. The MLE fitting uses both (7) and (9) as

(11) $$\left[\hat{\alpha },\hat{\beta },\hat{\sigma }\right]=\underset{}{\arg}\min_{\alpha ,\beta ,\sigma }\left\{-\left(LLF+LLF^{*}\right)\right\}.$$

Therefore, the only difference is whether the censored data are used or not. In Section 7, the influence of noise-limited PL in model fitting is then examined by comparing positioning error statistics using the OLS fitted model and MLE fitted model.

It should be noted that, e.g., in the case of a very small ratio of σ/α or if only very short link distances exist in the training data, then the OLS and MLE fitted models can be practically identical. In practice, in many typical radio channel conditions, there is a wide range of link distances where there is a relatively large probability of censored PL.

Path loss-based positioning can be done also without the training phase. In that case, the PL model parameters can be taken, e.g., from a standard channel model. The OLS fitting result can be seen as serving double duty, both as the OLS fitting result and as a (rather poor) example of an educated guess in the absence of training data.

## 5. Model Fitting Results

The single-slope log distance PL model (1) and (2) was fitted to training data. The training data were created using the PL distribution defined in the three examples. The model fitting results are presented and analyzed in Section 5.1, Section 5.2 and Section 5.3. In all these examples, for simplicity, the training data were created assuming omnidirectional antennas at both ends of the link and with a large sample size. Large sample size is needed to avoid uncertainty in the parameter estimates [5,10]. Additionally, a uniform distribution of distances was used [11,40].

### 5.1. Example 1

**Figure 3:**
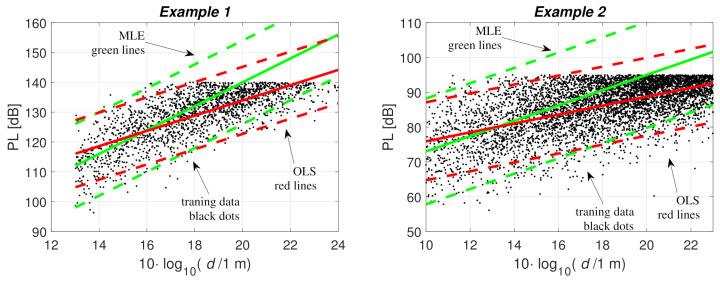
Fitting the path loss model to the training data with OLS (green) and MLE (red)—Example 1 (α=4, β=60, σ=7) and Example 2 (α=2.2, β=51). Training data with PL under the noise threshold are shown with black dots, solid lines show $\bar{PL}\left(d\right)$, and the dash lines are $\bar{PL}\left(d\right)\pm 1.96\hat{\sigma }$.

In this example the true PL distribution is the log-distance PL model (1) and (2) with the parameters α=4, β=60 dB, σ=7 dB, and noise threshold 140 dB [13]. The PL distribution, threshold level, and the fitted models are illustrated in Figure 3. Note that the figure shows only samples of measurable PL data points but the link distances and the number of noise-limited censored PL data points are also known.

Training data were created with a uniform distribution of distances between 20 and 500 m. The PL model was fitted to the data using OLS or Tobit MLE. With OLS the noise-limited censored data are ignored and with MLE all data are taken into account. The fitted PL models are illustrated in Figure 3 and the parameter estimates are listed in Table 1. The OLS fitting underestimates PL distribution for large link distances because there are more censored samples above the true mean, and as they were omitted the expected value was lowered. The OLS fitting gives parameter estimates $\hat{\alpha }\approx$ 2.5, $\hat{\beta }\approx$ 83 dB, and $\hat{\sigma }\approx$ 5.7 dB. The MLE gives parameter estimates $\hat{\alpha }\approx$ 4, $\hat{\beta }\approx$ 60 dB, and $\hat{\sigma }\approx$ 7 dB. The MLE estimates are very close to the true values α= 4, β= 60 dB, and σ= 7 dB. As can be seen, ignoring the censored samples can lead to significant errors in the PL model distribution. The same conclusion was made in [10,11], and in this paper, we study the effects on positioning accuracy.

The noise threshold level $PL^{*}$ limits the maximum path loss that can be measured. The expected PL reaches the 140-dB level at a link distance of 100 m. Therefore, PL at that distance has a 50% probability of being immeasurable, i.e., censored. Due to the large shadow fading, σ=7 dB, there is significant probability of censored PL between 40 and 220 m, i.e., where the dash lines $\bar{PL}\left(d\right)\pm 1.96\sigma$ cross the noise threshold of 140 dB in Figure 3. The OLS and MLE fitted models give the same expected PL at a link distance of about 37 m. Therefore, for most reasonable link distances the OLS model underestimates the expected PL, i.e., overestimates the link distance for a given PL. With MLE, the link distance corresponding to $\bar{PL}=PL^{*}$ is 100 m and with the OLS fitted model, it is about 170 m. The OLS fitted model predicts a significant probability of censored PL between 63 and 490 m ($\bar{PL}\left(d\right)\pm 1.96\sigma$).

### 5.2. Example 2

In Example 2, the PL parameters for the single-slope log-distance PL model were taken from a measurement campaign [10]. In [10], the MLE parameter estimates are $\hat{\alpha }=2.2$, $\hat{\beta }=51$, and $\hat{\sigma }=7.6$, and we used these parameters as the true values. The noise threshold is $PL^{*}=95$ dB. The OLS fitting without the censored data gives $\hat{\alpha }=1.3$, $\hat{\beta }=63$, and $\hat{\sigma }=5.6$ [10]. The fitted PL models are illustrated in Figure 3; the training data were created with a uniform distribution of distances between 10 and 200 m. The parameter estimates are summarized in Table 1.

The true PL distribution and the MLE fitted model predict a significant probability of censored PL between 21 and 470 m ($\bar{PL}\left(d\right)\pm 1.96\sigma$). The OLS fitted model overestimates this range to 430–2200 m link distance range. This overestimation is clearly larger than in Example 1 due to smaller path loss exponent α.

### 5.3. Example 3

The 3GPP TR 38.901 channel model includes PL models for various scenarios from 0.5 to 100 GHz [14]. In this paper, we use this model for the urban microcell scenario at 2 and 28 GHz frequencies. The model describes the PL distributions for LOS and NLOS and the LOS probability model.

The LOS probability depends on the link distance:

(12) $$Pr_{\mathrm{LOS}}=\left\{\begin{array}{cc} \; & 1\;,\;d<18\;m\\ \; & \frac{18\;m}{d}+\exp\left(-\frac{d}{36\;m}\right)\left(1-\frac{18\;m}{d}\right)\;,\;18\;m<d. \end{array}\right.$$

The LOS probability is 100% up to 18 m, 50% at 52 m, 10% at 189 m, and 1% at 1800 m. In LOS, the expected PL is

(13) $${\bar{PL}}_{\mathrm{LOS}}=\left\{\begin{array}{cc} \; & PL_{1}\;,\;18\;m<d<d_{\mathrm{BP}}^{\prime }\\ \; & PL_{2}\;,\;d_{\mathrm{BP}}^{\prime }<d<5000\;m \end{array}\right.$$

(14) $$PL_{1}=32.4+21\log_{10}\left(d\right)+20\log_{10}\left(f_{c}\right)$$

(15) $$PL_{2}=32.4+40\log_{10}\left(d\right)+20\log_{10}\left(f_{c}\right)-9.5\log_{10}\left({\left(d_{\mathrm{BP}}^{\prime }\right)}^{2}+{\left(h_{\mathrm{BS}}-h_{{}_{\mathrm{UT}}}\right)}^{2}\right),$$

where $f_{c}$ is frequency in GHz, $h_{\mathrm{BS}}$ is the base station height, $h_{\mathrm{UT}}$ is the user terminal height, and $d_{\mathrm{BP}}^{\prime }$ is the break point defined as [14]

(16) $$d_{\mathrm{BP}}^{\prime }=4h_{\mathrm{BS}}^{\prime }h_{\mathrm{UT}}^{\prime }f_{c}/c_{0},$$

where $h_{\mathrm{BS}}^{\prime }=h_{\mathrm{BS}}-1$ m and $h_{\mathrm{UT}}^{\prime }=h_{\mathrm{UT}}-1$ m, $f_{c}$ is the frequency in Hz, and $c_{0}$ is the speed of light. We assume $h_{\mathrm{BS}}=10$ m and $h_{\mathrm{UT}}=2$ m. The breakpoint distances are 240 and 3400 m at 2 and 28 GHz, respectively. Therefore, within reasonable link distances, at 2 GHz the LOS PL model is a dual-slope model with path loss exponent 2.1 up to 240 m and 4.0 for longer link distances.

In NLOS, the expected PL is

(17) $${\bar{PL}}_{\mathrm{NLOS}}=\max\left({\bar{PL}}_{\mathrm{LOS}},{\bar{PL}}_{\mathrm{NLOS}}^{\prime }\right),$$

where

(18) $${\bar{PL}}_{\mathrm{NLOS}}^{\prime }=35.3\log_{10}\left(d\right)+22.4+21.3\log_{10}\left(f_{c}\right)-0.3\left(h_{\mathrm{UT}}-1.5\;m\right),$$

and ${\bar{PL}}_{\mathrm{LOS}}$ is given by (13). The NLOS PL model has a path loss exponent of 3.53 and stronger frequency dependency than the LOS model.

The shadow fading is modeled as a zero-mean log-normal distribution with σ=4 dB in LOS and σ=7.82 dB in NLOS.

**Figure 4:**
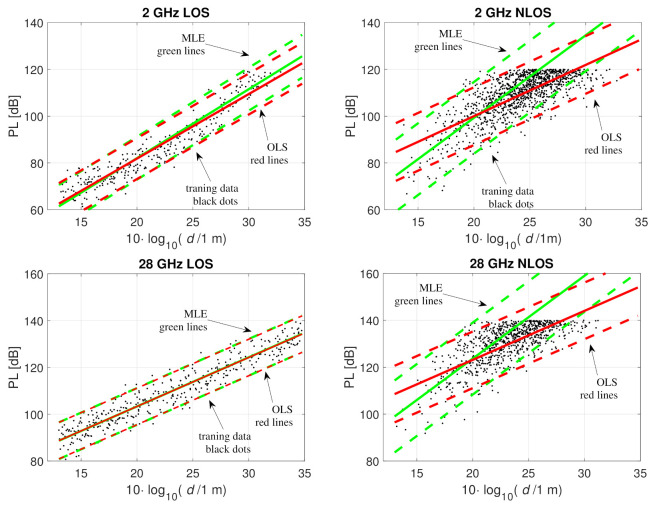
Fitting the path loss model to the training data with OLS (green) and MLE (red); Example 3, 2 GHz LOS, 2 GHz NLOS, 28 GHz LOS, 28 GHz NLOS. Training data with PL under the noise threshold are shown with black dots, solid lines show $\bar{PL}\left(d\right)$, and the dash lines are $\bar{PL}\left(d\right)\pm 1.96\hat{\sigma }$.

Training data were created with a uniform distribution of distances between 20 and 3000 m and divided into LOS and NLOS according to the LOS probability. The training data, fitted models, and noise threshold levels are illustrated in Figure 4 for LOS and NLOS at 2 and 28 GHz. Noise threshold levels of 120 and 140 dB were used at 2 and 28 GHz, respectively. The parameter estimates are summarized in Table 2. In LOS, censored PL, with the chosen $PL^{*}$ and maximum link distance range, is rare, and therefore, the OLS and MLE fitted models are quite similar. In NLOS, there is a clear difference between OLS and MLE, just as in Examples 1 and 2 presented in Section 5.1 and Section 5.2.

Again, let us look at where the fitted models cross the noise threshold level. At both frequencies the MLE fitted model for NLOS matches the true PL distribution at $\bar{PL}=PL^{*}$ and $\bar{PL}\left(d\right)\pm 1.96\sigma =PL^{*}$. These distances are 140, 390, and 1100 m at the 2 GHz frequency and 110, 290, and 800 m at 28 GHz. Similarly to examples 1 and 2, the OLS fitted model overestimates these as 220, 820, and 3000 m in the 2 GHz case and as 170, 640, and 2400 m at 28 GHz. The accuracy of the model at the threshold level becomes relevant when these models are used in the positioning phase to define likelihood functions for BSs with $PL>PL^{*}$.

## 6. Censored Path Loss and Positioning

Path loss model-based positioning is based on getting distance estimates from measured PL to BSs with known locations. For a given distance the PL model describes a probability distribution for PL, or inversely a PL value gives a distribution for the link distance. The width of the distribution is proportional to shadow fading. In ordinary trilateration positioning (OTP), distance estimates to contacted BSs are used to trilaterate the positioning estimate.

When fitting the model to training data, the censored PL is a measurement result at a known link distance and PL known to be more than the noise threshold level. In positioning, censored PL is an uncontacted BS due to PL larger than the threshold. Therefore, the minimum link distance has a probability distribution associated with the noise threshold level. Thus, failing to contact a given BS has position information as it means that the true position is unlikely to be close to that particular BS. Figure 1 illustrates examples of likelihood functions associated with measured PL and censored PL. Next, we will frame the positioning problem as a Tobit maximum likelihood estimation (MLE) in a similar manner as in the case of fitting the PL model to training data.

Let us first write the log-distance as $q=10\cdot \log_{10}\left(d/d_{0}\right)$, then the expected distance is

(19) $$\bar{q}\left(PL_{i},\theta_{i}\right)=\left(PL_{i}-\beta +G\left(\theta_{i}\right)\right)/\alpha ,$$

(20) $$q^{*}\left(\theta_{i}\right)=\left(PL^{*}-\beta +G\left(\theta_{i}\right)\right)/\alpha ,$$

where $PL_{i}$ is the measured PL to *i*th base station (or beam), $PL^{*}$ is the noise threshold, $q^{*}$ is the corresponding log-distance, and the standard deviation corresponding to shadow fading is σ/α. $q_{i}$ is the log-distance from point (x,y) to BS${}_{i}$ (or beam) and $\theta_{i}$ is the beam offset angle.

The log-likelihood function for known measured PL at point (x,y) is

(21) $$LLF=\sum_{i=1}^{N_{BS}}\left(-\ln\frac{\sigma }{\alpha }+\ln\phi \left(\frac{q_{i}-\bar{q}\left(PL_{i},\theta_{i}\right)}{\sigma /\alpha }\right)\right),$$

where $N_{BS}$ is the number of BSs (or directive beams) with measured PL under the noise threshold. The log-likelihood function for censored PL at point (x,y) is

(22) $$LLF^{*}=\sum_{i=1}^{N_{BS}^{*}}\ln\left(1-\Phi \left(\frac{q^{*}\left(\theta_{i}\right)-q_{i}}{\sigma /\alpha }\right)\right),$$

where $N_{BS}^{*}$ is the number of BSs (or directive beams) with censored PL. The position estimate $\left(\hat{x},\hat{y}\right)$ is derived as the minimum of the negative of the log-likelihood function. For the OTP positioning, the censored samples are not used; i.e.,

(23) $$\left[\hat{x},\hat{y}\right]=\underset{}{\arg}\min_{x,y}\left\{-LLF\right)\}.$$

The MLE positioning uses both (21) and (22) as

(24) $$\left[\hat{x},\hat{y}\right]=\underset{}{\arg}\min_{x,y}\left\{-\left(LLF+LLF^{*}\right)\right\}.$$

Thus, the only difference is whether or not the location information from the noise-limited censored PL data is used. In Section 7, the influence of noise-limited PL in model fitting is then examined by comparing positioning error statistics using the OLS fitted model and MLE fitted model.

Far away, uncontacted BSs have no effect on the positioning result, and therefore, it is useful to limit the BSs that are considered. In principle, the considered BSs should be chosen based on the properties of the likelihood functions, i.e., based on the PL model. In practice, of course, the database of known BS locations is also limiting which BSs can be considered. In this work, we propose to limit the considered censored BSs to an area around the OTP result with a radius equal to the link distance where the PL model predicts $\bar{PL}\left(d\right)-1\cdot \sigma =PL^{*}$. This limit was chosen due to a small performance difference observed between selection criteria $\bar{PL}\left(d\right)-1\cdot \sigma =PL^{*}$ and $\bar{PL}\left(d\right)-2\cdot \sigma =PL^{*}$. Importantly, the OLS and MLE fitted models give different radii for the area. The OLS fitted models systematically overestimate the link distances related to $PL^{*}$, and therefore, OLS-MLE includes many more censored BSs than MLE-MLE.

## 7. Positioning Simulation Results

The four options, with and without the censored data, were compared by conducting simulations in a regular BS grid, following a hexagonal layout. The true PL values were calculated based on the three examples, and positioning simulation results are presented and analyzed in Section 7.1, Section 7.2 and Section 7.3. The positioning is based on PL model derived, in Section 5.1, Section 5.2 and Section 5.3, either by OLS or by MLE. Positioning is done either with OTP or with Tobit MLE, as presented in Section 6. Therefore, the four options are OLS-OTP, MLE-OTP, OLS-MLE, and MLE-MLE. Positioning error is the distance between the true position and the estimated position. These statistics are based on 10,000 samples with random true locations and different realizations of SF. In all examples, two BS antennas are considered, omni-directional (N=1) and directional beams with eighth beams (N=8 and $\theta_{3\mathrm{dB}}=45^{\circ }$). A few BS grid densities are considered where the distance between BSs, $d_{\mathrm{BS}}$, is selected such that an average number of contacted BSs, ${\bar{N}}_{\mathrm{BS}}$, is, e.g., 5.0. It is assumed that the signals from the BSs do not interfere and the BS locations and beam directions are known.

**Figure 5:**
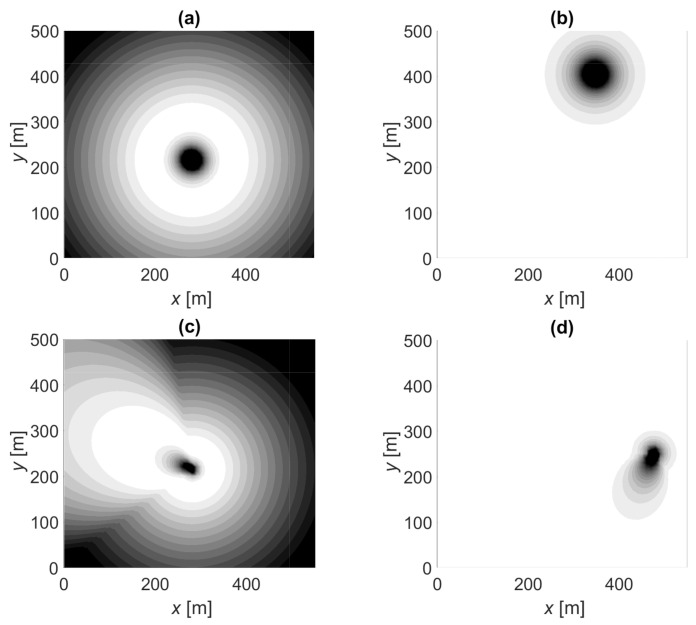
Likelihood function illustrations: (**a**) measured PL and omnidirectional antenna, (**b**) censored PL and omnidirectional antenna, (**c**) measured PL and directive beam (N=8), (**d**) censored PL and directive beam (N=8). White is likely, gray is possible, and black is an unlikely location.

**Figure 6:**
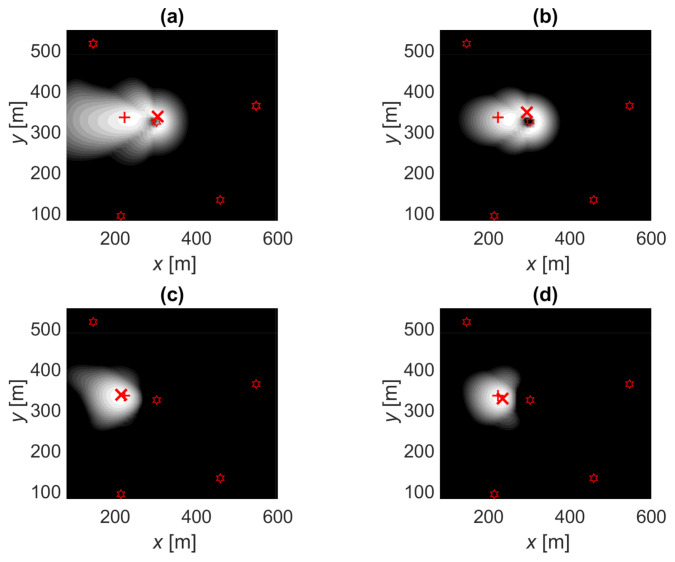
Likelihood function illustrations: true location (+), position estimate (×), base stations (BSs; stars), contact with three BSs with directive antennas (N=8) at $\left(x_{\mathrm{BS}},y_{\mathrm{BS}}\right)$ = (302,324), (547,363), and (146,516). White is likely, gray is possible, and black is an unlikely location.(**a**) OLS-OPT: ordinary least squares fitting and trilateration positioning. (**b**) MLE-OPT: Tobit MLE fitting and the ordinary trilateration positioning. (**c**) OLS-MLE: ordinary least squares fitting and the Tobit MLE positioning. (**d**) MLE-MLE: Tobit MLE fitting and the Tobit MLE positioning.

Before we analyze the positioning error statistics, let us look at the likelihood function illustrations presented in Figure 5 and Figure 6. These figures were calculated assuming that the true PL follows the distribution in Example 1 and positioning using OLS and MLE fitted models presented in Section 5.1. In Figure 5a is the $LLF_{i}$ of a single contacted BS with an omnidirectional antenna where the highest likelihood is found on a ring around the BS. In Figure 5b is the $LLF_{i}^{*}$ of an uncontacted BS showing a low likelihood close to the BS. Figure 5c,d shows the corresponding examples with single directive antennae where the likelihood functions are stretched by the antenna gain pattern. Figure 6 shows an example of the sum of the likelihood functions in one location using the four options. In this example, the distance between BSs is 245 m, BSs antennas have eight beams (N=8, $\theta_{3\mathrm{dB}}=45^{\circ }$), and three BSs are contacted with a total of four beams ($PL<PL^{*}$). The positioning errors are 81, 72, 9, and 14 m for OLS-OTP, MLE-OTP, OLS-MLE, and MLE-MLE, respectively. Using MLE, positioning results in smaller and narrower likelihood function optimum. Let us compare OLS-OTP, Figure 6a, and OLS-MLE, Figure 6c. The OTP result is to the right-hand side to the true location, and the the closest uncontacted BSs are on the right side of the OTP result at $\left(x_{\mathrm{BS}},y_{\mathrm{BS}}\right)=\left(385,521\right)$ and (430,123). In MLE positioning these BSs are taken into account, which corrects the positioning away from the uncontacted BSs and thus reduces the error. Thus, MLE positioning can, at least sometimes, compensate for the error resulting from the OLS fitted model, which overestimates the link distances.

### 7.1. Example 1

**Figure 7:**
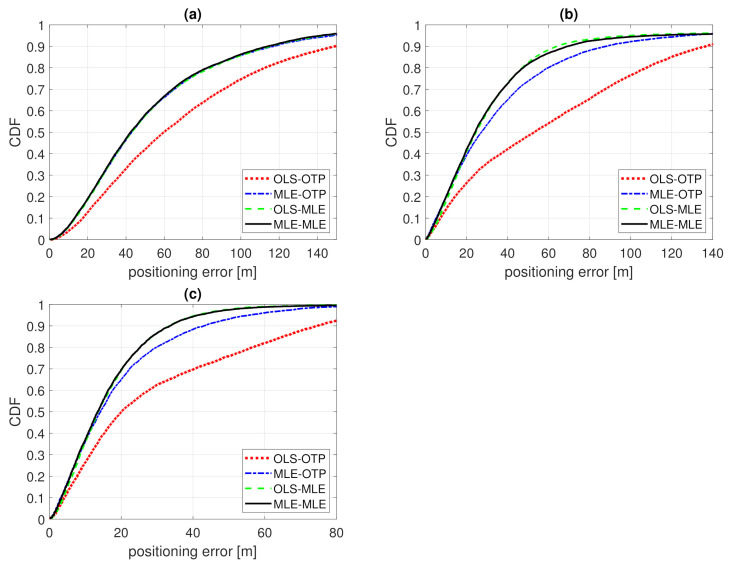
CDF of positioning error of Example 1. Model fitting to training data with ordinary least squares (OLS-) or Tobit MLE fitting (MLE-) and positioning with either ordinary trilateration (-OTP) of Tobit MLE positioning (-MLE). (**a**) N=1, ${\bar{N}}_{\mathrm{BS}}=2$; (**b**) N=8, ${\bar{N}}_{\mathrm{BS}}=2$; (**c**) N=8, ${\bar{N}}_{\mathrm{BS}}=5$.

In this section, we analyze the positioning results assuming the true PL follows the Example 1 distribution. The OLS and MLE model fitting results are presented in Section 5.1 and positioning is based on (4) with parameters from Table 1 and $PL^{*}=140$ dB. Three BS grid densities are considered, leading to an average of 2, 3, or 5 contacted BSs. Each average number of contacted BSs, ${\bar{N}}_{\mathrm{BS}}$, corresponds to a constant distance between neighbours $d_{\mathrm{BS}}$. Two BS antennas are considered, omni-directional and directional beams with N=8 and $\theta_{3\mathrm{dB}}=45^{\circ }$. Positioning error 50th and 90th percentiles are listed in Table 3 and three of the error CDFs are presented in Figure 7.

The considered BSs with censored PL are limited to an area around the OTP result with a radius equal to the link distance where the PL model predicts $\bar{PL}\left(d\right)-1\cdot \sigma =PL^{*}$. This radius depends on the PL model. With the MLE fitted model, this is 150 m and with the OLS it is 300 m. The OLS fitted model overestimates the large link distances, and therefore, more censored BSs are taken into account with OLS-MLE than with the MLE-MLE. For example, in the case of ${\bar{N}}_{\mathrm{BS}}=5$ and N=8, OLS-MLE includes an average of 13 censored BSs and MLE-MLE includes only an average of 2.9. Note that in this example the positioning error statistics do not change significantly even if more censored BSs are included. When comparing OLS-MLE and MLE-MLE there are two effects. First, the OTP positioning based on OLS fitted model creates typically larger error than the OTP positioning based on MLE fitted model. Second, a larger number of censored BSs accounted for in case of OLS-MLE may correct the error caused by positioning with OLS fitted model, whereas in case of MLE-MLE a smaller number of censored BSs being included may be sufficient.

The BS grid density affects the positioning accuracy. In a regular hexagonal grid, the distance between BSs is constant. The positioning error with the directive antennas is smaller than with the omnidirectional antennas, and similarly, with the denser BS grid the errors are smaller. These trends apply to all the presented cases and also for the other examples of the true PL distribution.

In all of the simulated cases, OLS-OTP is the worst. It is the worst-case scenario with the PL model that does not fit the reality and has positioning that does not use all the available information. For example, with omnidirectional antennas and ${\bar{N}}_{\mathrm{BS}}=2$, the median errors are 60, 43, 43, and 42 m for OLS-OTP, MLE-OTP, OLS-MLE, and MLE-MLE, respectively. In this case, there is a 29% improvement from OLS-OTP to MLE-MLE.

Using MLE in either phase improves accuracy. The best example of this is a dense BS grid with ${\bar{N}}_{\mathrm{BS}}=5$ and the directive antennas resulting in the 90th percentile error of 74 m with OLS-OTP and a 42–55% improvement to about 43–33 m with MLE in either or both phases (see Table 3). When using the noise-limited censored PL with MLE in both phases, MLE-MLE is in general the most accurate option. The improvement from OLS-OTP to MLE-MLE ranged from 29% (from 60 to 42 m with *N* = 1 and ${\bar{N}}_{\mathrm{BS}}$ = 2) to 55% (from 53 to 24 m with *N* = 8 and ${\bar{N}}_{\mathrm{BS}}$ = 2) for the median error and from 24% (from 149 to 114 m with *N* = 1 and ${\bar{N}}_{\mathrm{BS}}$ = 2) to 56% (from 101 to 45 m with *N* = 8 and ${\bar{N}}_{\mathrm{BS}}$ = 3) for the 90th percentile.

Lastly, let us compare MLE-OTP to OLS-MLE. The median and the 90th percentiles are close to each other. In MLE-OTP, the positioning is based on the correct PL model but the censored PL is not used in the positioning phase. In OLS-MLE, the censored PL is used in the positioning phase with MLE, but the OLS fitting results in wrong parameter estimates, as shown in Section 4. As pointed out earlier, the OLS fitting result can be also interpreted as (a rather poor) educated guess in the absence of training data. Using MLE in the positioning phase compensates for the poor PL model. Therefore, it can be concluded that if the noise-limited censored PL is taken into account in the positioning phase, the training phase is perhaps not needed, or at least, the training phase is not very critical when MLE positioning is used.

### 7.2. Example 2

**Figure 8:**
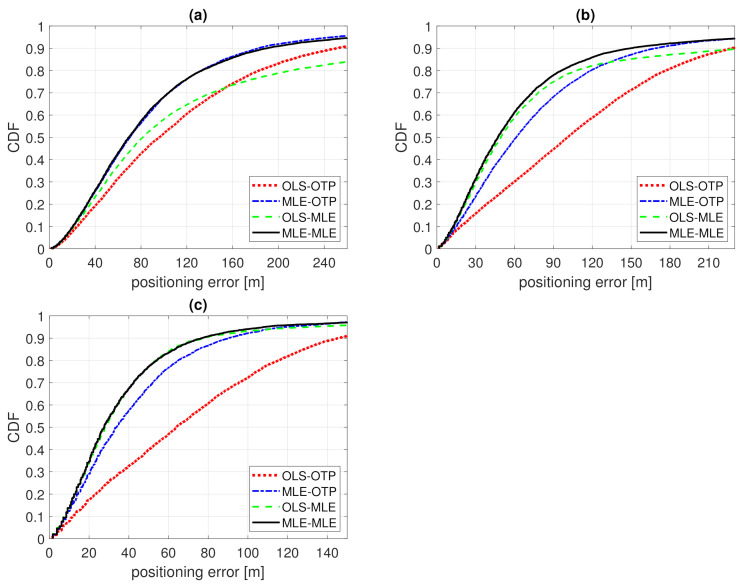
CDF of positioning error of Example 2. Model fitting to training data with ordinary least squares (OLS-) or Tobit MLE fitting (MLE-) and positioning with either ordinary trilateration (-OTP) of Tobit MLE positioning (-MLE). (**a**) N=1, ${\bar{N}}_{\mathrm{BS}}=5$; (**b**) N=8, ${\bar{N}}_{\mathrm{BS}}=5$; (**c**) N=8, ${\bar{N}}_{\mathrm{BS}}=10$.

In this section, we analyze the positioning results while assuming the true PL follows Example 2’s distribution. The OLS and MLE model fitting results are presented in Section 5.2 and positioning is based on (4) with parameters from Table 1 and $PL^{*}=95$ dB. Two BS grid densities are considered, leading to an average of five or ten contacted BSs. Two BS antennas are considered, omni-directional and directional beams with N=8 and $\theta_{3\mathrm{dB}}=45^{\circ }$. Positioning error 50th and 90th percentiles are listed in Table 4 and three of the error CDFs presented in Figure 8. The BSs with censored PL around the OTP results, OLS-OTP and MLE-OTP, are included if they lie withing 85 and 220 m in case of OLS-MLE and MLE-MLE, respectively. For example, in the case of ${\bar{N}}_{\mathrm{BS}}=5$ and N=8, OLS-MLE includes an average of 21 censored BSs and MLE-MLE includes only an average of 1.2.

The relative performances of OLS-OTP, MLE-OTP, OLS-MLE, and MLE-MLE are the same as in Example 1. Using MLE in either case reduces the positioning errors; OLS-OTP is the worst and MLE-MLE is the best method. For example, in the case of ${\bar{N}}_{\mathrm{BS}}=5$ and omnidirectional BS antennas, the improvement in median error is 35% or 28% for Examples 1 and 2, respectively. The errors are larger in Example 2 than in Example 1 due to larger σ/α, i.e., larger standard deviation of the expected log-distance (19). The improvement from OLS-OTP to MLE-MLE ranged from 28% (from 96 to 69 m with *N* = 1 and ${\bar{N}}_{\mathrm{BS}}$ = 5) to 57% (from 64 to 28 m with *N* = 8 and ${\bar{N}}_{\mathrm{BS}}$ = 10) for the median error and from 24% (from 251 to 190 m with *N* = 1 and ${\bar{N}}_{\mathrm{BS}}$ = 5) to 47% (from 146 to 77 m with *N* = 8 and ${\bar{N}}_{\mathrm{BS}}$ = 10) for the 90th percentile.

It can be noted that in this example, the tail end of the error CDF of OLS-MLE is lower than in any other result in this paper. For example, with ${\bar{N}}_{\mathrm{BS}}=5$ and N=1 the 90th percentile error is 352 m. This shows that occasionally, with a small number of contacted BSs (and large σ/α), MLE positioning can cause larger errors when the PL model does not fit the true distribution. In this example, the OLS fitted model overestimates the large link distances more than in Examples 1 and 3. In this example, because the link distance overestimation is so large, it could be beneficial to include more censored BSs in the case of OLS-MLE. For example, in case of ${\bar{N}}_{\mathrm{BS}}=5$ and N=1, the OLS-MLE 90th percentile error is 246 m with a 2300 m radius ($\bar{PL}\left(d\right)-2\cdot \sigma =PL^{*}$) and an average of 760 censored BSs around the OLS-OTP result. Apparently, the MLE positioning may help only if the overestimation in the link distances is not excessively large.

### 7.3. Example 3

In this section, we analyze the positioning results assuming the true PL follows the Example 3 distribution, given in Section 5.3, at 2 and 28 GHz. The OLS and MLE model fitting results for LOS and NLOS are presented in Section 5.3. The positioning is based on (4) with parameters from Table 2 and $PL^{*}=120$ dB and 140 dB at 2 and 28 GHz, respectively. The noise threshold is varied for one 2 GHz case to test the performance with different cutoff values. The LOS probability (12) is used to determine the LOS/NLOS state for the BSs. For simplicity, we assume that the LOS detection probability is 100% and the appropriate LOS or NLOS model parameters are used. BSs with censored PL are assumed to be in NLOS. In practice, the LOS state needs to be detected [41,42,43]. One 28 GHz case is presented with 100%, 95%, 90%, 85%, and 80% LOS detection rates in the positioning phase. LOS detection error leads to using LOS model parameters in NLOS, or NLOS parameters in LOS, and therefore, severe overestimation or underestimation of the link distances, respectively.

Two BS grid densities are considered, leading to an average of five or ten contacted BSs, and two BS antennas are considered, omnidirectional and eight directional beams with N=8 and $\theta_{3\mathrm{dB}}=45^{\circ }$. The numbers of detected LOS and NLOS BSs depend on the average total number of contacted BSs, BS antenna, frequency, and the noise threshold. For example, ${\bar{N}}_{\mathrm{BS}}=10$ and the eight-sector directional antennas (N=8) the average number of contacted LOS BSs is 1.2 or 2.1 at 2 and 28 GHz, respectively. At 2 GHz, BSs with censored PL around the OTP results are included if they lie within 1600 and 640 m (OLS-MLE and MLE-MLE, respectively). At 28 GHz, these numbers are 1300 and 480 m. For example, at 2 GHz in the case of ${\bar{N}}_{\mathrm{BS}}=5$ and N=8, OLS-MLE includes an average of 17 censored BSs and MLE-MLE includes an average of only 2.2.

**Figure 9:**
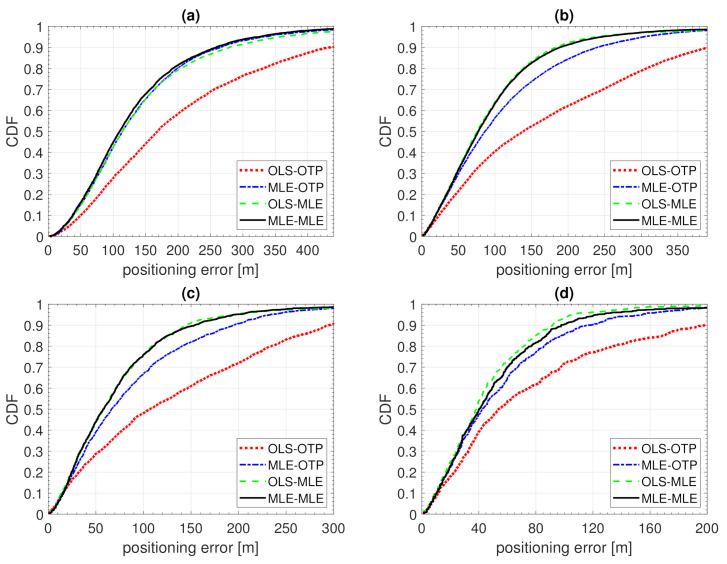
CDF of positioning error of Example 3. Model fitting to training data with ordinary least squares (OLS-) or Tobit MLE fitting (MLE-) and positioning with either ordinary trilateration (-OTP) of Tobit MLE positioning (-MLE). (**a**) 2 GHz, N=1, ${\bar{N}}_{\mathrm{BS}}=5$, (**b**) 2 GHz, N=8, ${\bar{N}}_{\mathrm{BS}}=5$, (**c**) 28 GHz, N=8, ${\bar{N}}_{\mathrm{BS}}=5$, (**d**) 28 GHz, N=8, ${\bar{N}}_{\mathrm{BS}}=10$.

Positioning error 50th and 90th percentiles are listed in Table 5 and Table 6, and four of the error CDFs are presented in Figure 9. At 2 GHz, the improvement from OLS-OTP to MLE-MLE ranged from 27% (from 73 to 53 m with *N* = 8 and ${\bar{N}}_{\mathrm{BS}}$ = 10) to 44% (from 139 to 77 m with *N* = 8 and ${\bar{N}}_{\mathrm{BS}}$ = 5) for the median error and from 40% (from 434 to 259 m with *N* = 1 and ${\bar{N}}_{\mathrm{BS}}$ = 5) to 52% (from 392 to 188 m with *N* = 8 and ${\bar{N}}_{\mathrm{BS}}$ = 5) for the 90th percentile. At 28 GHz, the improvement from OLS-OTP to MLE-MLE ranged from 25% (from 54 to 40 m with *N* = 8 and ${\bar{N}}_{\mathrm{BS}}$ = 10) to 46% (from 107 to 58 m with *N* = 8 and ${\bar{N}}_{\mathrm{BS}}$ = 5) for the median error and from 36% (from 394 to 253 m with *N* = 1 and ${\bar{N}}_{\mathrm{BS}}$ = 5) to 51% (from 198 to 98 m with *N* = 8 and ${\bar{N}}_{\mathrm{BS}}$ = 10) for the 90th percentile.

Even though the underlying PL model is quite different than in Examples 1 and 2, the influence of the noise-limited PL is the same. Usually the error percentiles in Table 3, Table 4, Table 5 and Table 6 are lower for OLS-MLE than MLE-OPT. This indicates that including the censored PL is more critical in the positioning phase than in the training phase. Of course, if possible, the MLE-MLE method should be used when possible. The influence of including the noise-limited PL in the positioning phase is much greater from OLS-OTP to OLS-MLE compared to MLE-OTP to MLE-MLE. In other words, including the censored PL in positioning is especially effective for correcting or compensating for the difference between the OLS fitted model and the true PL distribution. When the PL model fits well, the influence of MLE positioning is clearly smaller and can be mostly seen in the 90th percentiles of MLE-OTP and MLE-MLE. For example, at 28 GHz with N=8 and ${\bar{N}}_{\mathrm{BS}}=5$, the 90th percentile error is reduced by 21% from MLE-OTP to MLE-MLE.

Positioning error 50th and 90th percentiles are listed in Table 7 for noise cutoff levels varying from 120 dB to 140 dB at 2 GHz, N=1, and ${\bar{N}}_{\mathrm{BS}}=$ 5. Since the average number of contacted BSs is fixed, the higher threshold levels lead to larger distance between the BSs. With larger $PL^{*}$, and larger $d_{\mathrm{BS}}$, the errors are larger but quite stable when compared to $d_{\mathrm{BS}}$. For example, with MLE-MLE the median error is a constant 24% of the $d_{\mathrm{BS}}$. The improvement from OLS-OTP to MLE-MLE ranged from 29% (from 554 to 391 m with $PL^{*}=140$ dB) to 36% (from 234 to 150 m with $PL^{*}=125$ dB) for the median error and from 33% (from 1396 to 929 m with $PL^{*}=140$ dB) to 41% (from 595 to 351 m with $PL^{*}=125$ dB) for the 90th percentile. Therefore, performance improvement by using MLE is not sensitive to the noise cutoff level.

Positioning error 50th and 90th percentiles are listed in Table 8 for positioning-phase LOS detection probabilities $P_{\mathrm{pos}}$. The detection probability is varied from 100% to 80% at 28 GHz, N=8, and ${\bar{N}}_{\mathrm{BS}}=$ 10. For simplicity, detection probability is 100% in the training phase and same PL model parameters can be used. LOS detection errors cause larger positioning errors since wrong PL model parameters are used. With worse $P_{\mathrm{pos}}$ the relative improvement from OLS-OTP to MLE-MLE increases for the median but decreases for the 90th percentile. The improvement from OLS-OTP to MLE-MLE ranged from 25% (from 54 to 40 m with $P_{\mathrm{pos}}$ 100%) to 38% (from 126 to 78 m with $P_{\mathrm{pos}}$ 80%) for the median error and from 23% (from 548 to 421 m with $P_{\mathrm{pos}}$ 80%) to 51% (from 198 to 98 m with $P_{\mathrm{pos}}$ 100%) for the 90th percentile. Therefore, the results demonstrate improved positioning performance when the noise-limited censored PL is taken into account also in the case of LOS detection errors.

## 8. Conclusions

In this paper, we have shown that the noise-limited censored PL data can be used in the training and positioning phases of PL model-based positioning. The censored data, i.e., when PL is larger than the noise threshold, can be taken into account using Tobit MLE when fitting the model to the training data and also in the positioning phase.

Three different PL distribution examples are used as the true PL. Simulations compared PL model fitting and positioning results both without and with the noise-limited PL data. The results show that if the censored PL is omitted in the training phase, then the fitted PL model overestimates the long link distances. It is also shown that when the censored PL is taken into account, the fitted model matches the true distribution well. Positioning simulations were conducted with a simple log-distance law PL model. The results show improved positioning accuracy when the censored PL is properly taken in to account with MLE. A selection criterion based on PL model properties was presented that limits the number of BSs in the MLE positioning. The positioning error median and 90th percentile were reduced by 23% to 57% when MLE is used in both phases as compared to when it is omitted in both. Positioning error reductions were demonstrated in a wide range of radio channel properties. These included different path loss exponents, omnidirectional and directional BS antennas, and different BS grid densities. Additionally, separate distributions for LOS and NLOS, various noise threshold values, and LOS detection probabilities were considered. The results also indicate that if the censored PL is taken into account in the positioning phase, then the accuracy of the PL model fitting to training data becomes far less important. With the improved accuracy and robustness against PL model fitting errors, the PL-based positioning show good promise, especially if combined with other high-precision positioning and tracking methods.

## Figures and Tables

**Table 1:** Fitted ordinary least squares (OLS) and maximum likelihood estimation (MLE) parameter estimates for Example 1 (α=4, β=60, σ=7) and Example 2 (α=2.2, β=51, σ=7.6).

		$\hat{\alpha }$	$\hat{\beta }$	$\hat{\sigma }$
Example 1	OLS	2.5	83	5.8
	MLE	4.0	60	7.0
Example 2	OLS	1.3	63	5.6
	MLE	2.2	51	7.6

**Table 2:** Fitted OLS and MLE parameter estimates for Example 3 at 2 and 28 GHz.

			$\hat{\alpha }$	$\hat{\beta }$	$\hat{\sigma }$
2 GHz	LOS	OLS	2.8	27	4.5
		MLE	3.0	23	4.7
	NLOS	OLS	2.2	56	6.3
		MLE	3.5	29	7.8
28 GHz	LOS	OLS	2.1	61	4.0
		MLE	2.1	61	4.0
	NLOS	OLS	2.1	81	6.2
		MLE	3.5	53	7.8

**Table 3:** Positioning error, in meters, 50th and 90th percentiles of Example 1. Base stations have omnidirectional (N=1) or directional antennas (N=8) and the average number of contacted BSs is ${\bar{N}}_{\mathrm{BS}}=$ 2, 3, or 5.

${\bar{N}}_{\mathrm{BS}}$	2		3		5		2		3		5	
*N*	1		1		1		8		8		8	
$d_{\mathrm{BS}}$	159		129		100		245		199		154	
	50%	90%	50%	90%	50%	90%	50%	90%	50%	90%	50%	90%
OLS-OTP	60	149	43	108	29	77	53	137	34	101	20	74
MLE-OTP	43	117	29	72	20	48	27	88	19	59	14	43
OLS-MLE	43	116	28	72	20	44	25	64	19	44	14	33
MLE-MLE	42	114	28	69	19	43	24	69	18	45	13	34

**Table 4:** Positioning error, in meters, 50th and 90th percentiles of Example 2. Base stations have omnidirectional (N=1) or directional antennas (N=8) and the average number of contacted BSs is ${\bar{N}}_{\mathrm{BS}}=$ 5 or 10.

${\bar{N}}_{\mathrm{BS}}$	5		10		5		10	
*N*	1		1		8		8	
$d_{\mathrm{BS}}$	459		325		515		420	
	50%	90%	50%	90%	50%	90%	50%	90%
OLS-OTP	96	251	66	164	101	228	64	146
MLE-OTP	71	182	46	105	61	168	34	90
OLS-MLE	82	352	49	259	49	236	28	78
MLE-MLE	69	190	45	106	47	149	28	77

**Table 5:** Positioning error, in meters, 50th and 90th percentiles of Example 3 (2 GHz) with variable noise threshold level $PL^{*}$. Base stations have omni-directional (*N* = 1) BS antennas and the average number of contacted BSs is ${\bar{N}}_{\mathrm{BS}}=$ 5.

$PL^{*}$	120 dB		125 dB		130 dB		135 dB		140 dB	
$d_{\mathrm{BS}}$	459 m		623 m		846 m		1164 m		1600 m	
	50%	90%	50%	90%	50%	90%	50%	90%	50%	90%
OLS-OTP	168	434	234	595	316	801	425	1063	554	1396
MLE-OTP	114	265	158	377	218	525	302	716	416	987
OLS-MLE	114	279	154	361	206	500	283	683	395	950
MLE-MLE	110	259	150	351	203	485	283	674	391	929

**Table 6:** Positioning error, in meters, 50th and 90th percentiles of Example 3 (28 GHz) with variable LOS detection probability in the positioning phase $P_{\mathrm{pos}}$. Base stations have directional antennas (N=8) and the average number of contacted BSs is ${\bar{N}}_{\mathrm{BS}}=$ 10.

$P_{\mathrm{pos}}$	100		95		90		85		80	
	50%	90%	50%	90%	50%	90%	50%	90%	50%	90%
OLS-OTP	54	198	64	258	78	357	98	458	126	548
MLE-OTP	42	117	50	183	61	270	74	341	96	436
OLS-MLE	38	92	41	126	47	196	55	288	66	403
MLE-MLE	40	98	45	148	53	244	63	329	78	421

**Table 7:** Positioning error, in meters, 50th and 90th percentiles of Example 3 (2 GHz). Base stations have omnidirectional (N=1) or directional antennas (N=8) and the average number of contacted BSs is ${\bar{N}}_{\mathrm{BS}}=$ 5 or 10.

${\bar{N}}_{\mathrm{BS}}$	5		10		5		10	
*N*	1		1		8		8	
$d_{\mathrm{BS}}$	253		207		160		113	
	50%	90%	50%	90%	50%	90%	50%	90%
OLS-OTP	168	434	104	273	139	392	73	250
MLE-OTP	114	265	73	162	86	240	57	156
OLS-MLE	114	279	73	158	76	184	50	119
MLE-MLE	110	259	70	150	77	188	53	132

**Table 8:** Positioning error, in meters, 50th and 90th percentiles of Example 3 (28 GHz). Base stations have omnidirectional (N=1) or directional antennas (N=8) and the average number of contacted BSs is ${\bar{N}}_{\mathrm{BS}}=$ 5 or 10.

${\bar{N}}_{\mathrm{BS}}$	5		10		5		10	
*N*	1		1		8		8	
$d_{\mathrm{BS}}$	390		276		565		399	
	50%	90%	50%	90%	50%	90%	50%	90%
OLS-OTP	158	394	97	250	107	295	54	198
MLE-OTP	110	258	68	160	66	195	42	117
OLS-MLE	109	275	66	146	59	144	38	92
MLE-MLE	107	253	64	144	58	154	40	98

## Data Availability

Not applicable.
